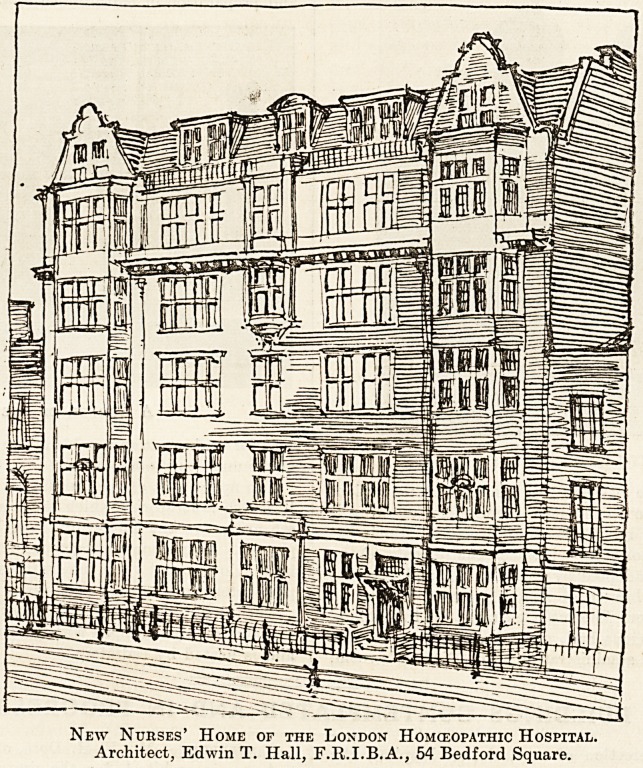# The Homœopathic Hospital New Nurses' Home

**Published:** 1911-06-03

**Authors:** 


### THE HOMŒOPATHIC HOSPITAL NEW NURSES' HOME.

ARCHITECT'S DESCRIPTION AND OPENING CEREMONY.

On Tuesday last week the Duchess of Hamilton and Brandon visited the London Homœopathic Hospital and laid the commemoration stone of the new nurses' home in what will be the entrance hall. A bouquet was presented to the Duchess by the Matron while Mr. Edwin T. Hall, the architect, presented a silver trowel, with the help of which the Duchess laid the stone. The following is Mr. Hall's description of the building, the final appearance and elevation of which are well represented by the accompanying drawing.

The new home for nurses, which faces the Homœopathic Hospital building proper, is now about half-built, and it is hoped will be ready for occupation by the end of the year. It is a building seven storeys in height, the principal elevation being of red brick with Portland stone dressings, the design being an English domestic Renaissance.

The ground floor plan shows at the west end a sitting-room for nurses, in the centre a sisters' sitting-room, and at the east end a large sitting-room for probationers. On the south side is the Home sister's office, and there are four bedrooms on this floor. In the basement there is a large gymnasium for nurses, maids' sitting-room, heating chamber, and scullery. On the upper floors there are separate bedrooms for seventy nurses, and in the sanitary tower at the back are placed all the w.c.s, housemaids' closets, bathrooms, etc., while additional bathrooms are placed on the upper floors near the staircase. In addition to the main staircase there is a fire-escape staircase at the south-east end. An electric lift is carried from the bottom to the top of the building.

The construction is of fire-resisting materials throughout, the floors being of reinforced concrete. The roof, also fire resisting in construction, is covered with Broseley tiles, the top being flat and affording intercommunication between the two staircases. The dining-rooms for the staff are in a separate building contiguous to the Hospital kitchen. The contractors are Messrs. Prestige and Co., and the Clerk of the Works is Mr. Cornes.

New Nurses' Home of the London Homœopathic Hospital.
Architect, Edwin T. Hall, F.R.I.B.A., 54 Bedford Square.

In accordance with the practice which has made the files of The Hospital practically a complete architectural record of hospital construction, we hope to publish the plans of the Home in facsimile. When they have been redrawn to scale for this purpose, and submitted to expert opinion, they will complete in detail the general outline we have given this week as current news. It should be added that King Edward's Hospital Fund has made a grant of £500 towards the cost of the building.

## Figures and Tables

**Figure f1:**